# Retraction note: A histopathological analysis of the epidemiology of coronary atherosclerosis: an autopsy study

**DOI:** 10.1186/s13000-016-0558-3

**Published:** 2016-11-02

**Authors:** Negin Abedinzadeh, Behnam Pedram, Yasan Sadeghian, Seyed Mohamad Hossein Tabatabaei Nodushan, Maryam Gilasgar, Mahsa Darvish, Aram Mokarizadeh

**Affiliations:** 1 Graduate, Faculty of Medicine, Tehran University of Medical Sciences, Tehran, Iran; 2 Department of Pathobiology, Susangerd Branch Islamic Azad University, Susangerd, Iran; 3 Graduate, Faculty of Medicine, Shahid Beheshti University of Medical Sciences and Health Services, Tehran, Iran; 4 Cellular & Molecular Research Center, and Department of Immunology, Kurdistan University of Medical Sciences, Sanandaj, Iran

## Retraction

The Editor-in-Chief and Publisher have retracted this article [1] because the scientific integrity of the content cannot be guaranteed. An investigation by the Publisher found it to be one of a group of articles we have identified as showing evidence suggestive of attempts to subvert the peer review and publication system to inappropriately obtain or allocate authorship. This article showed evidence of plagiarism (most notably from the articles cited [2–7]) and authorship manipulation.
